# Potential Association Between Vaping and Recurrent Aphthous Stomatitis: A Case Series

**DOI:** 10.7759/cureus.70238

**Published:** 2024-09-26

**Authors:** Anusha Kotha, Surendra Reddy Mandapati, Hemanth Rudraraju

**Affiliations:** 1 Dentistry, Blunn Creek Family Dentistry, Austin, USA; 2 Dentistry, Advanced Dental Care, East Hartford, USA; 3 Dentistry, CaliDental, Santa Maria, USA

**Keywords:** acute aphthous stomatitis, case series, e-cigarettes, electronic cigarettes, e-smoking, health risks of e-cigarettes and vaping, oral mucosa lesions, vaping

## Abstract

Since their invention, e-cigarettes have gained significant popularity. However, they are relatively new, and researchers are still striving to understand the long-term and comprehensive effects of vaping on the body. The impact of vaping on the oral cavity, in particular, remains underexplored. This case series aims to address this gap by examining patients with a history of vaping who presented with aphthous ulcers.

In this case series, we describe three adults aged 30 to 45 years who came in with the chief complaint of ulcers on the oral mucosa. All patients were regular or social vapers. Notably, their lesions resolved with little to no intervention. This observation prompts further exploration into vaping as a potential etiological factor for recurrent aphthous stomatitis (RAS).

## Introduction

Recurrent aphthous stomatitis (RAS), also known as canker sores, ranks among the most prevalent oral diseases globally [1,2]. Clinical presentations often feature recurrent bouts of singular or multiple, shallow, round/ovoid, painful ulcers with well-defined margins. These lesions are covered by a grey-white pseudo membrane and erythematous haloes of varying sizes. Lesions less than 1 cm in size are called minor RAS and those that exceed 1 cm in diameter are called major RAS [3,4]. Etiological factors contributing to these lesions are numerous, including local trauma, psychological stress, autoimmune conditions, genetic, hematological disorders, and viral infections [2,5]. Minor RAS usually heal within 10 to 14 days, while major RAS lesions take up to six weeks to heal. Minor RAS lesions heal without scarring, but major RAS leave scars after healing [3]. In terms of treatment, there has been no singular treatment that has been deemed effective, and there is inconclusive evidence regarding the optimal systemic intervention for RAS [1,2,5,6].

E-cigarettes emerged in the 2000s as an alternative to cigarettes and have become popular among both smokers and non-smokers. Since their emergence, new information about the effects of vaping on oral health is emerging daily [7]. Some small-scale, cross-sectional studies have raised concerns about dryness, irritation, and gum diseases, ultimately leading to oral ulceration seen with vaping [8]. However, interpreting these data is challenging due to the rapid emergence of new vaping products and the cross-sectional nature of the studies [9].

This case series examines the incidence of RAS in patients who smoke e-cigarettes. Three known vapers were included in the study after obtaining informed consent and detailed medical, oral, and social histories. These patients frequently reported experiencing RAS on the labial mucosa and the lateral border of the tongue.

## Case presentation

Case 1

**Figure 1:**
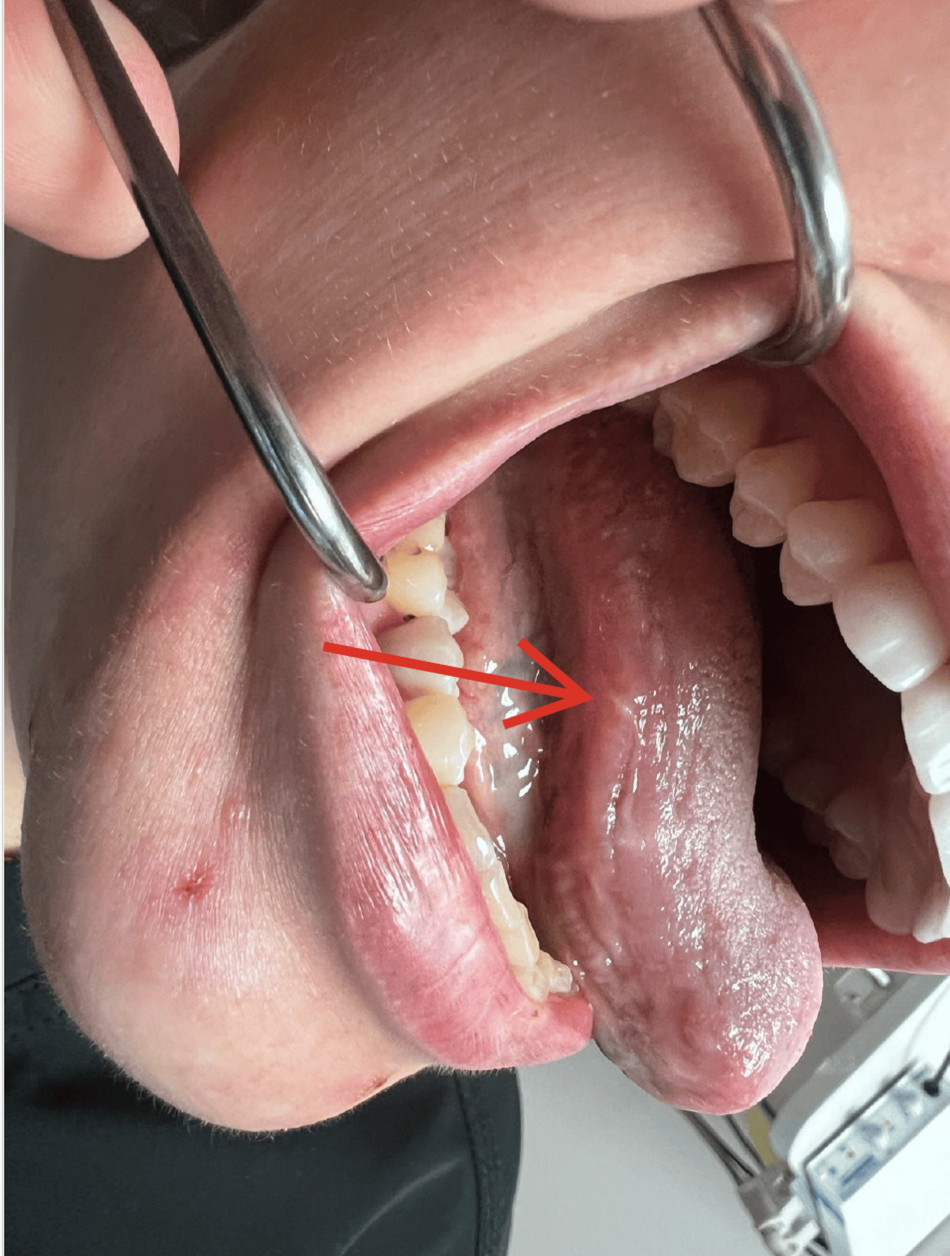
Case 1 - Ulceration on the lateral border of the tongue.

A 35-year-old female with hypothyroidism managed with medication and a history of tobacco use presented with recurrent episodes of aphthous stomatitis that began after she started using e-cigarettes in 2017. There was no evidence of local trauma, psychological stress, autoimmune conditions, genetic factors, hematological disorders, or viral infections. The patient had a 10-year history of cigarette smoking and a two-year history of dipping tobacco and switched to e-cigarettes in 2017 as part of her effort to quit smoking and chewing tobacco. She reported using the e-cigarette every few hours, sometimes multiple times per hour. Since then, she has experienced oral ulcers almost monthly for the past six years, primarily affecting the lateral tongue (Figure 1), inner cheek, and labial mucosa.

The ulcers, ranging from one to four in number and measuring 3 x 5 mm, typically resolved within three to five days without the need for medical intervention.

Case 2

**Figure 2:**
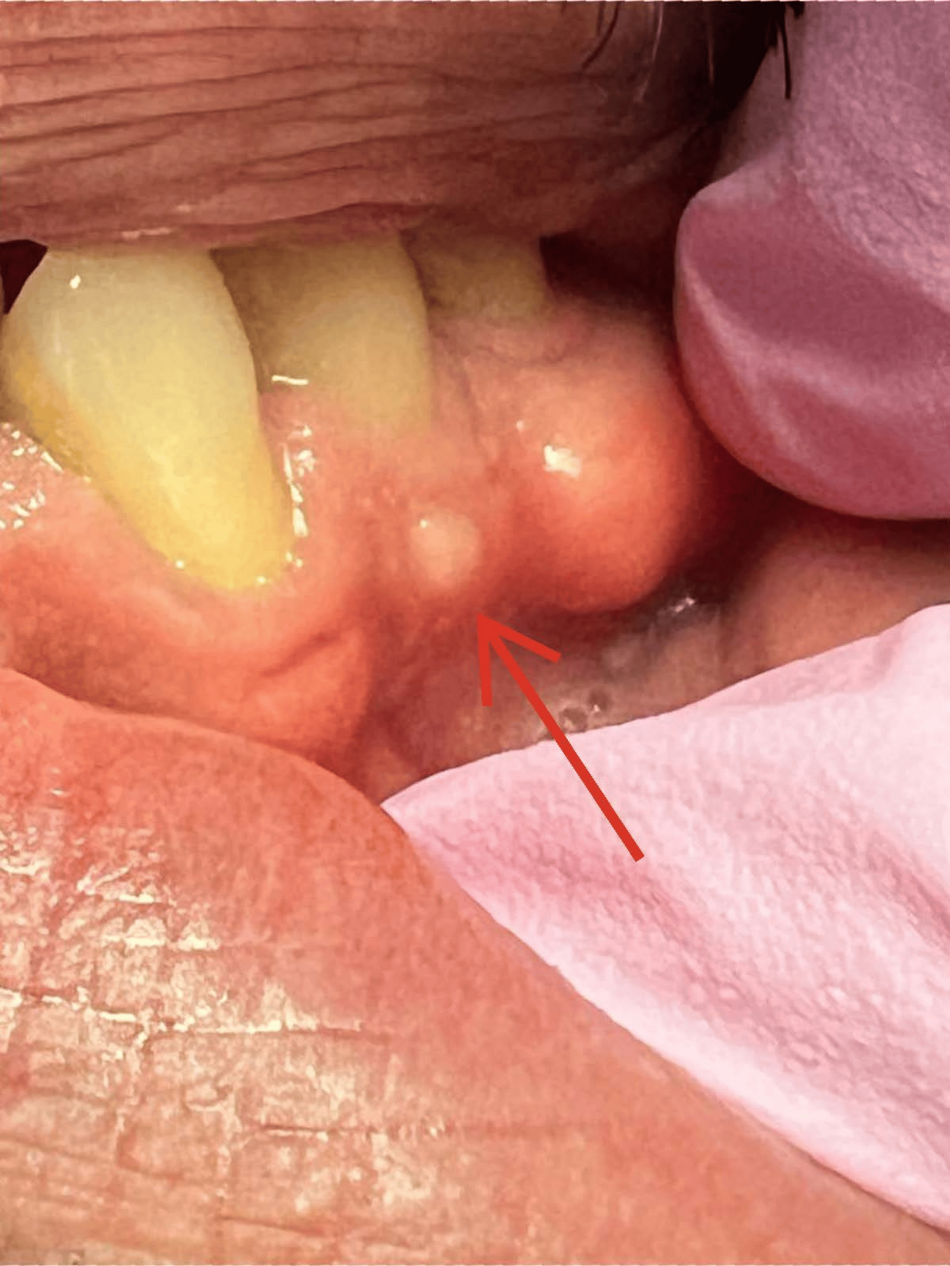
Case 2 - Ulceration on the attached gingiva.

A 30-year-old male with no significant medical history and not taking any medication presented with recurrent ulceration of the labial mucosa and gingiva. There was no evidence of local trauma, psychological stress, autoimmune conditions, genetic factors, hematological disorders, or viral infections. The patient had a four-year history of e-cigarette usage, which coincided with the onset of the ulcers. These 1-2 mm in diameter ovoid ulcers appeared on the attached gingiva and buccal mucosa (Figure 2) and occurred once or twice a month, resolving spontaneously without medical intervention.

The patient used Orabase for symptomatic relief. Prior to e-cigarette use, the patient had no history of oral ulcers. After discontinuing e-cigarette use, the ulcers stopped recurring. No additional diagnostic tests were performed, as the condition resolved following cessation of e-cigarette usage.

Case 3

**Figure 3:**
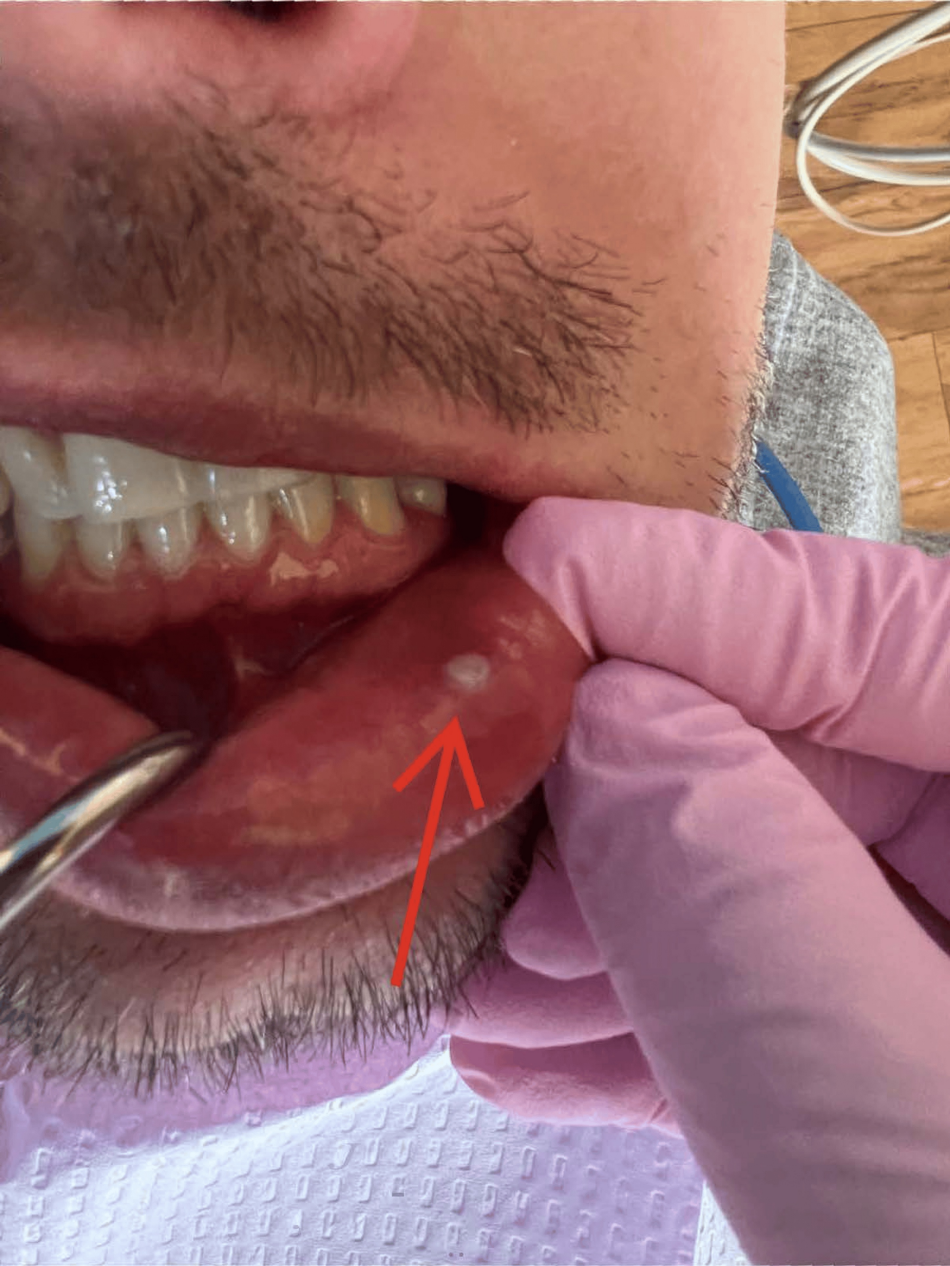
Case 3 - Ulceration on the labial mucosa.

A 42-year-old male with a 10-year history of hypertension, controlled without medication, presented with recurrent oral ulcerations. There was no evidence of local trauma, psychological stress, autoimmune conditions, genetic factors, hematological disorders, or viral infections. The patient reported using e-cigarettes for five years, which coincided with the onset of recurrent ulcerations on the labial mucosa. He discontinued e-cigarette use a year ago and had a history of social marijuana use. The oral ulcers occurred one to two times per month and typically resolved spontaneously without treatment. However, in March 2023, the patient experienced a severe outbreak of RAS with significant pain lasting four days, prompting a visit to a dental office. The ulcers were localized on the labial mucosa (Figure 3) and resolved within five days.

For pain relief, the patient was prescribed a topical corticosteroid and anesthetic, which alleviated the pain, and the ulcers healed. Since discontinuing e-cigarette use, the patient has not experienced any further episodes of aphthous ulcers, suggesting a potential link between e-cigarette use and the occurrence of RAS.

## Discussion

Recurrent aphthous ulcers (RAUs) have been linked to various conditions, though definitive etiological factors have yet to be conclusively established. No single cause has been proven to directly result in these ulcers. However, these cases suggest a potential association between vaping and the development of aphthous ulcers.

E-cigarettes are still the subject of ongoing research, with new findings continuously emerging since their introduction. Some studies [8,10] have observed a correlation between e-cigarette use and symptoms such as dryness of the mouth and ulceration. The chemicals found in e-cigarettes, including aldehydes, heavy metals, carbonyls, and flavoring chemicals, can cause cytotoxic effects and inflammatory responses. This evolving body of research highlights the need for further investigation into the health impacts of e-cigarettes, including their possible role in causing RAUs.

There is no reliable standard treatment protocol for RAUs. Previous studies [3,4] suggest the use of chlorhexidine gluconate solution and benzydamine hydrochloride mouthwash for therapeutic relief. Topical tetracycline can be used to reduce the severity of ulceration; however, it does not prevent recurrence of the lesions. In addition, topical corticosteroids, including hydrocortisone and triamcinolone, are the most commonly used medication to treat RAUs but they do not reduce the recurrence of the lesions. Moreover, systemic medications such as colchicine, pentoxifylline, steroids, dapsone, thalidomide, and pidotimod are used in the form of systemic therapy. Laser therapy has been found to successfully reduce pain, accelerate healing time, and decrease both the number and size of ulcers. Systemic glucocorticoids have also been successful in treating major RAS.

Findings can help dental healthcare providers, enabling them to offer better advice and care to patients who vape. Increased awareness among vapers about the potential risk of developing aphthous ulcers can encourage preventive measures and prompt them to seek early treatment. The study could pave the way for additional research on the long-term effects of vaping on oral health, leading to a more comprehensive understanding of its impacts.

## Conclusions

RAUs may be another potential side effect of vaping. Although the precise cause of these ulcers is not fully understood, this study's findings indicate a possible connection between e-cigarette use and the development of recurring oral ulcers. This underscores the need for additional research to fully explore the range of health impacts linked to vaping, particularly regarding oral health. Expanding our understanding in this area will enable us to offer more informed guidance and recommendations to e-cigarette users, helping to reduce potential health risks.

## References

[1] Staines K, Greenwood M (2015). Aphthous ulcers (recurrent). BMJ Clin Evid.

[2] Shah K, Guarderas J, Krishnaswamy G (2016). Aphthous stomatitis. Ann Allergy Asthma Immunol.

[3] Porter SR, Scully C, Pedersen A (1998). Recurrent aphthous stomatitis. Crit Rev Oral Biol Med.

[4] Akintoye SO, Greenberg MS (2014). Recurrent aphthous stomatitis. Dent Clin North Am.

[5] Brocklehurst P, Tickle M, Glenny AM (2012). Systemic interventions for recurrent aphthous stomatitis (mouth ulcers). Cochrane Database Syst Rev.

[6] Randall DA, Wilson Westmark NL, Neville BW (2022). Common oral lesions. Am Fam Physician.

[7] Yang I, Sandeep S, Rodriguez J (2020). The oral health impact of electronic cigarette use: a systematic review. Crit Rev Toxicol.

[8] Rouabhia M (2020). Impact of electronic cigarettes on oral health: a review. J Can Dent Assoc.

[9] Holliday R, Chaffee BW, Jakubovics NS, Kist R, Preshaw PM (2021). Electronic cigarettes and oral health. J Dent Res.

[10] Ebersole J, Samburova V, Son Y (2020). Harmful chemicals emitted from electronic cigarettes and potential deleterious effects in the oral cavity. Tob Induc Dis.

